# Experiment and Simulation Study of Wheel Angle on the Ultra-Precision Scribing Quality of LCD Glass Panels

**DOI:** 10.3390/mi17060650

**Published:** 2026-05-25

**Authors:** Jinzhu Guo, Xijing Zhu, Yongjin Wang, Yao Liu

**Affiliations:** 1 Shanxi Key Laboratory of Semiconductor Ultraprecision Machining Technology and Intelligent Equipment, North University of China, Taiyuan 030051, China; 18234146352@163.com (J.G.); 15530081973@163.com (Y.W.); 2 CETC Fenghua Information-Equipment Co., Ltd, Taiyuan 030062, China

**Keywords:** SPH simulation, scribing wheel angle, median crack depth, scribing force, effective stress

## Abstract

To investigate the effect of scribing wheel angle on the scribing behavior of LCD glass, an SPH-based numerical model was established in LS-DYNA and validated against experimental results for reaction force and median crack depth. The results show that the model can accurately capture the mechanical response and crack propagation during the scribing process. At a scribing depth of 10 μm, the maximum relative errors between simulation and experiment were 5.17% for reaction force and 2.36% for median crack depth. The results for the 110° scribing wheel indicate that median cracks mainly initiate and propagate rapidly during the penetration stage, while the median crack depth becomes nearly stable after the preset depth is reached, and the subsequent rolling stage has little influence on further crack growth. As the wheel angle increases from 90° to 140°, the experimental mean peak reaction force increases from 2.66 N to 9.97 N, the maximum effective stress increases from 374.4 MPa to 732.8 MPa, and the median crack depth increases from 68 μm to 97 μm. Experimental observations further show that small wheel angles tend to cause debris accumulation and edge chipping, whereas excessively large wheel angles are likely to induce lateral cracks. Overall, a wheel angle of about 110° provides better cross-sectional quality, surface quality, and crack controllability for 0.2 mm-thick LCD glass.

## 1. Introduction

Liquid crystal displays (LCDs) are widely used in consumer electronics, automotive displays, industrial control systems, and intelligent terminals because of their mature technology, low cost, and stable performance [1]. In addition to conventional display manufacturing, LCD-related glass substrates and panel materials are also increasingly relevant to semiconductor packaging, microelectronic modules, and advanced optoelectronic devices [2,3]. With the rapid development of chip packaging, panel-level integration, microelectronic display modules, and advanced optoelectronic systems, increasingly stringent requirements have been imposed on the dimensional accuracy, edge integrity, and subsurface damage control of brittle glass substrates. As a result, the high-quality scribing and separation of LCD glass are no longer limited to display production, but are also closely related to precision manufacturing for semiconductor-supporting and microelectronic components.

**Figure 1:**
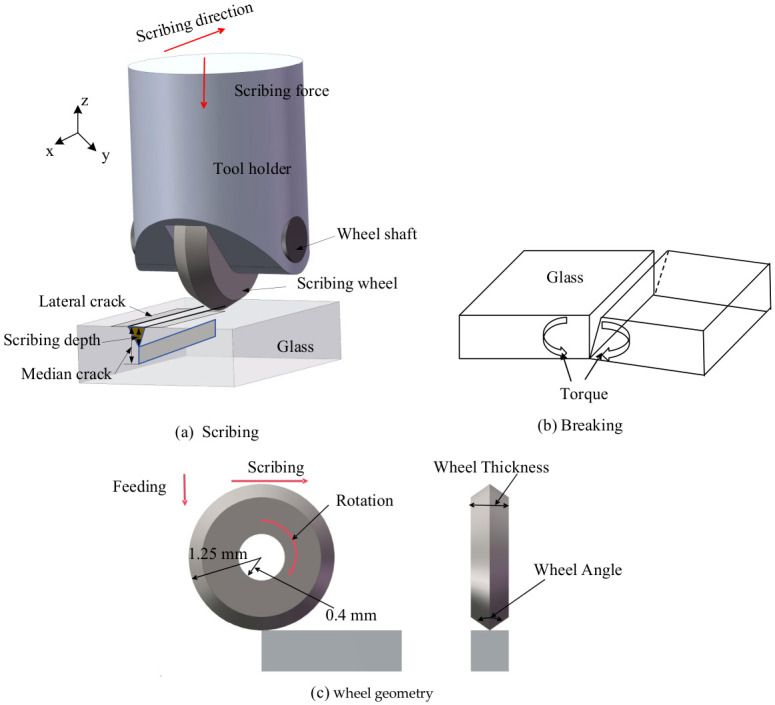
Glass panel separation process: (**a**) scribing; (**b**) breaking; (**c**) wheel geometry.

High-quality separation of LCD glass substrates is a key step in display manufacturing. Current processing methods for display glass mainly include wheel scribing [4], laser non-destructive cutting [5], water-jet cutting [6], ultrafast laser cutting [7], and hot-wire thermal-stress cutting [8]. Among them, wheel scribing is still the most widely used method because of its mature process, low cost, high efficiency, strong industrial applicability, and suitability for controlled high-quality separation [9]. In this process, the wheel first forms a scribing line on the glass surface and generates surface cracks and median cracks [10], as shown in Figure 1a, and the glass is then separated by mechanical breaking, as shown in Figure 1b. However, these cracks and their propagation directly affect cut quality, edge strength, and the stability of the subsequent breaking process [11]. Therefore, wheel scribing of display glass is not only a conventional separation method, but also an important ultra-precision machining technique for thin brittle materials. In practical manufacturing, the scribing depth is usually controlled at the micrometer scale, and the morphology, depth, and stability of the induced cracks largely determine the final edge quality, dimensional accuracy, and breaking reliability. Here, “ultra-precision scribing” refers to the controlled scribing of 0.2 mm-thick LCD glass with a scribing depth of 10 μm. This process requires micro-scale control of wheel penetration, crack initiation, median crack depth, lateral crack formation, edge chipping, and cross-sectional uniformity to ensure stable breaking and high-quality panel edges. Accordingly, clarifying the influence of wheel parameters on crack initiation and propagation is of great significance for improving the machining quality of thin glass substrates.

At present, studies on crack control in wheel scribing are mainly experimental [12]. Chen et al. [13] reported that, for hard and high-fracture-toughness materials such as sapphire, a smaller wheel angle can reduce tensile stress and suppress lateral cracks. Müller-Braun [14] found that, in float glass scribing, a smaller wheel angle combined with lower scribing pressure can produce deeper median cracks and improve edge strength. Kong et al. [15] further showed that, for ultrathin glass and OLED panel glass, wheel angle significantly affects lateral crack width, median crack depth, and cross-sectional integrity, making it a key factor in scribing quality. Li Dawei et al. [16] also suggested that wheel selection for ultrathin display glass should consider glass thickness, hardness, scribing speed, and machining accuracy. They recommended a wheel angle of 105–115° and analyzed the formation of median and lateral cracks through experiments and simulations.

Although many experimental results have been reported, glass scribing involves highly transient brittle fracture. The initiation, propagation, and instability of median cracks often occur in a very short time, making them difficult to capture in real time with conventional methods. In recent years, techniques such as high-speed imaging, photoelastic observation, and fracture morphology analysis have provided new ways to study crack evolution. For example, Hasegawa et al. [17] studied fracture surface evolution during glass wheel scribing using high-speed photoelastic observation. Imai et al. [18] observed crack initiation and propagation dynamically. Murakami et al. [19] further investigated median crack repropagation after scribing and the formation of different crack surfaces. These studies show that crack propagation in wheel scribing is not a simple static damage process, but a dynamic process controlled by wheel geometry, contact load, local stress field, and material damage.

To overcome the limits of experiments, numerical simulation has become an important tool for studying crack propagation in glass. The finite element method is widely used to analyze crack growth and residual stress. For crack growth, the element deletion method and shared-node technique are commonly used [20], while residual stress analysis is often carried out using the extended finite element method (XFEM) [21]. In comparison, the smoothed particle hydrodynamics (SPH) method has unique advantages in dealing with large deformation, material fragmentation, and dynamic crack propagation because it is mesh-free [22]. It has already been used in single-grit scratching models and in studies of crack evolution in fused silica glass [23].

For this purpose, a 0.2 mm-thick LCD glass substrate was selected, and an SPH model coupled with the JH-2 constitutive model was established to study the transient mechanical response, damage evolution, and median crack propagation during wheel scribing. Compared with previous studies mainly focused on experimental observations of wheel-angle effects, this work validates the model using reaction force and median crack depth measurements, and further links wheel angle with stress distribution and crack evolution. The main contribution is to reveal the stage-dependent mechanism of crack formation: median cracks are mainly generated during penetration, while subsequent rolling primarily affects stress redistribution and contact-force evolution. These results provide guidance for wheel-angle selection and high-quality scribing of thin LCD glass.

## 2. Modelling of Scribing Process

### 2.1. The JH-2 Model for Brittle Material

In 1994, Johnson and Holmquist further developed the JH-2 model based on JH-1 by introducing continuous damage-induced strength degradation to describe the progressive failure of brittle materials. The JH-2 model considers the effects of strain rate and hydrostatic pressure, and incorporates a damage-dependent strength model together with a polynomial equation of state [24]. During loading, the material initially exhibits elastic behavior. Once the stress reaches the yield limit, damage begins to accumulate. As the damage evolves, the material progressively degrades and eventually fails completely. The JH-2 model consists of three components: strength, damage, and pressure [25].

The strength part describes the relationship between the strength of undamaged materials and broken materials under pressure and strain rate. The normalized equivalent stress of the strength model is expressed as:

(1) $$\sigma^{*}={\sigma_{i}}^{*}-D({\sigma_{i}}^{*}-{\sigma_{f}}^{*})$$

where $\sigma_{i}^{*}$ is the normalized equivalent stress of undamaged materials, $\sigma_{f}^{*}$ is the normalized equivalent stress of broken materials, and D is the material damage degree [26].

The expression of normalized equivalent stress ($\sigma^{*}$, $\sigma_{i}^{*}$, $\sigma_{f}^{*}$) is generally:

(2) $$\sigma^{*}=\sigma /\sigma_{\mathrm{HEL}}$$

In which σ is the true equivalent stress, and $\sigma_{HEL}$ is the equivalent stress at the Hugoniot Elastic Limit (HEL) of the material. The general form of the true equivalent stress is:

(3) $$\sigma =\sqrt{\frac{1}{2}\left[{\left(\sigma_{x}-\sigma_{y}\right)}^{2}+{\left(\sigma_{x}-\sigma_{z}\right)}^{2}+6{\left({\tau_{xy}}^{2}+{\tau_{xz}}^{2}+{\tau_{yz}}^{2}\right)}^{2}\right]}$$

where $\sigma_{x}$, $\sigma_{y}$, $\sigma_{z}$ are normal stresses, and, $\tau_{xy}$, $\tau_{xz}$, $\tau_{yz}$ are shear stresses.

The normalized stress expressions of undamaged materials and broken materials are:

(4) $$\left\{\begin{array}{c} {\sigma_{i}}^{*}=A{\left(P^{*}+T^{*}\right)}^{N}(1+C\ln\varepsilon^{*})\\ {\sigma_{f}}^{*}=B{\left(P^{*}\right)}^{M}(1+C\ln\varepsilon^{*}) \end{array}\right.$$

The A, B, C, M, N, T and ${\sigma^{*}}_{max}$ are parameters related to the material [27].

The damage part adopts the accumulation method, which is similar to the JH-1 and Johnson-Cook models, and its expression is as follows:

(5) $$\begin{array}{c} D=\sum \left(\Delta \varepsilon_{p}/{\varepsilon_{p}}^{f}\right)\\ {\varepsilon_{p}}^{f}=D_{1}(P^{*}+T^{*})D_{2} \end{array}$$

where $\Delta \varepsilon_{p}$ is the equivalent plastic strain of the material in one operation cycle, $\varepsilon_{p}^{f}$ is the plastic strain of the material broken under constant pressure p, and $D_{1}$, $D_{2}$ are material constants.

The pressure part can be derived from the hydrostatic pressure before material fragmentation occurs (D = 0):

(6) $$P=K_{1}\mu +K_{2}\mu^{2}+K_{3}\mu^{3}$$

$K_{1}$, $K_{2}$, $K_{3}$ are material constants, μ is the volume strain, ρ is the current density of the material, and $\rho_{0}$ is the reference density of the material. This model includes the influence of expansion or swelling of brittle materials due to the increase in additional pressure, that is:

(7) $$P=K_{1}\mu +K_{2}\mu^{2}+K_{3}\mu^{3}+\Delta P$$

ΔP is the pressure change. The pressure change is considered from the energy perspective, and the pressure change process is reflected in an incremental way, as shown in the following formula:

(8) $$\Delta P=-K_{1}\mu_{t}+\Delta t+\sqrt{{\left(K_{1}\mu_{t}+\Delta t+\Delta P\right)}^{2}+2\beta_{o}K_{1}\Delta U}$$

$\beta_{0}$ is the conversion coefficient of elastic energy loss into hydrostatic potential energy.

### 2.2. Glass Scribing Model

Based on the crack propagation and mechanical principles of glass during scribing-wheel dicing, a dynamic simulation model was established to investigate the influence of wheel angle on crack propagation. Due to the large deformation and brittle fracture involved in the process, only the local effective scribing region of the glass and the scribing-edge portion of the wheel were retained to improve computational efficiency.

The glass domain was set to 0.27 mm × 0.18 mm × 0.2 mm, covering the wheel-glass contact zone, near-surface stress concentration region, and median crack propagation region along the thickness direction. This truncated local domain is sufficient for comparing local damage and crack propagation under different wheel angles while reducing computational cost. To reduce boundary effects, the lateral boundaries were placed away from the main contact region, and boundary constraints were applied to suppress abnormal motion of SPH particles near the edges. Therefore, the model is used for local mechanism analysis and relative comparison among wheel angles, rather than for reproducing full long-distance industrial scribing.

**Figure 2:**
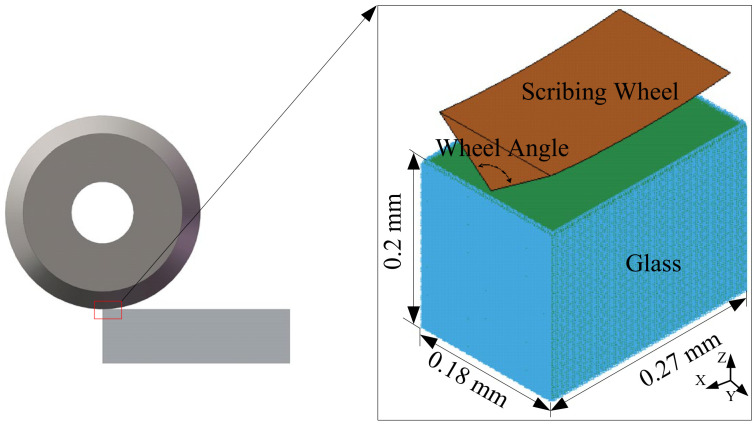
Simulation model.

The simplified model structure is shown in Figure 2. A scribing wheel with a diameter of 2.5 mm and an edge angle of 110° was first used for the simulation. Subsequently, wheels with different angles were modeled for comparative analysis. To compensate for deviations in mass and moment of inertia caused by simplifying the wheel geometry, the mass parameter TM and moments of inertia IXX, IYY, and IZZ were defined using PART_INERTIA. The simulation was conducted using LS-DYNA, and the model dimensions are shown in Figure 2.

### 2.3. Material Properties

The scribing wheel was discretized using a Lagrangian finite element mesh with a uniform element size of 9.6 µm, resulting in 1196 elements. This mesh size was selected to ensure stable contact calculation between the Lagrangian wheel elements and the SPH glass particles. A larger element size led to insufficient wheel-edge resolution and particle penetration at the wheel-glass interface, whereas the 9.6 µm mesh effectively reduced penetration and improved contact-force transmission.

For the glass sample of 0.27 mm × 0.18 mm × 0.2 mm, the SPH method was adopted to simulate large deformation, brittle fracture, and fragmentation during scribing. The particle spacing was determined through sensitivity analysis. A coarse spacing could not clearly resolve the crack tip or median crack morphology, while further refinement greatly increased the particle number and computational cost. Therefore, a spacing of 3 µm in the length and width directions and 2 µm in the thickness direction was adopted, generating 540,000 SPH particles and balancing crack-resolution capability with computational efficiency.

The scribing wheel was modeled as a rigid body using MAT_RIGID, with density, elastic modulus, and Poisson’s ratio defined according to typical alloy parameters, as listed in Table 1. Its y-direction displacement and rotations about the x- and z-axes were constrained to simulate actual motion restrictions.

The glass was modeled using the Johnson-Holmquist Ceramics (JH-2) model, which describes the strength, damage, and equation-of-state response of brittle materials under high-strain-rate loading. The basic material parameters, including density and shear modulus, were determined from the LCD glass properties and relevant literature. The JH-2 strength, damage, and equation-of-state parameters were mainly adopted from reported parameters for brittle glass-like materials [24,25]. Their applicability was evaluated by comparing simulated and experimental reaction force and median crack depth. The agreement indicates that these parameters can reasonably capture the response trend under the present scribing conditions, although further calibration may be required for other glass compositions, thicknesses, or loading conditions. The specific values are listed in Table 2 [26,27]. All parameters in Table 1 and Table 2 were converted consistently with the LS-DYNA input unit system; the density of 2.53 × 10^−6^ kg·mm^−3^ corresponds to 2530 kg·m^−3^.

### 2.4. Boundary Conditions and Loading

The scribing motion was precisely defined through kinematic parameters, with the entire process divided into two distinct phases. In the first phase, the alloy scribing wheel vertically penetrated the glass to a depth of 10 µm within 2 μs. In the second phase, after reaching the preset depth, the wheel performed a combined rolling and translational motion on the glass surface at a speed of 500 mm/s, with this phase lasting 8 μs. The total simulation time was set to 10 μs, including a 2 μs penetration stage and an 8 μs rolling stage. This time window was selected to capture the key local transient processes, including stress concentration, damage initiation, median crack formation, and early-stage rolling-induced stress redistribution. It should be noted that the selected time window is shorter than the full industrial scribing process. Therefore, the simulation results are mainly used to analyze the local crack-formation mechanism and the relative influence of wheel angle, rather than to directly represent the complete long-distance production scribing process.

Motion control was implemented using the DEFINE_CURVE keyword to define displacement-time and velocity-time curves, which were then applied to the rigid body of the alloy scribing wheel via BOUNDARY_PRESCRIBED_MOTION_RIGID. The interaction between the alloy scribing wheel and the glass was modeled using CONTACT_AUTOMATIC_NODES_TO_SURFACE, simulating the contact between the finite element nodes of the wheel and the SPH particles of the glass.

### 2.5. Output Variables and Post-Processing

To fully capture the fracture behavior during the LCD glass scribing process, this study set up multi-dimensional output indicators in the simulation to achieve refined data collection: through the DATABASE_EXTENT_BINARY module of the LS-DYNA R13.1.0 software, the damage history variables of SPH particles were output in a high-frequency mode, enabling dynamic tracking of the complete evolution process of glass materials from stress accumulation to failure and fracture; in the DATABASE_OPTIONZ module, the RCFORC parameter was selected to record the contact force data between the scribing wheel and the glass in real time, thus accurately obtaining the instantaneous change information of mechanical interactions during the scribing process; and for stress analysis, in the LS-PrePost 4.8.29 post-processing software, the “Von Mises stress” option was selected in the Fringe Component function bar to intuitively view and extract the distribution and numerical characteristics of Effective Stress. In the simulation data processing stage, the LS-PrePost 4.8.29 software was used to extract the original data of crack depth, reaction force and stress, and the data were subsequently imported into Origin 2024 software for statistical analysis and data visualization; through operations such as data fitting and trend analysis, the discrete simulation results were converted into quantitative crack depth values and reaction force variation curves, providing accurate data support for the subsequent mechanism analysis of the influence of scribing wheel angle on scribing performance.

## 3. Modelling Results and Discussions

### 3.1. Scribing Process

**Figure 3:**
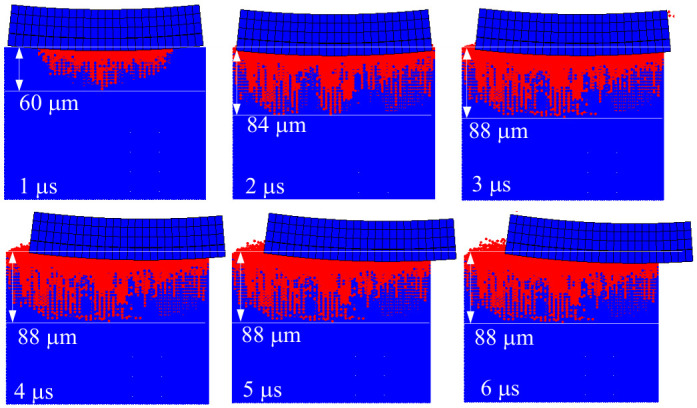
Scribing Process at 110°: Crack Depth (1–6 μs).

The dynamic process of the 110° scribing wheel—from initial penetration into the glass to final withdrawal—was comprehensively simulated. Figure 3 illustrates the scribing states at time intervals from t = 1 μs to t = 6 μs, capturing the evolution of both crack depth and reaction force output throughout this period.

During the vertical penetration of the scribing wheel into the glass in the Z-direction, median cracks were initiated and propagated rapidly upon initial contact. At 1 μs, the median crack depth had already reached 60 μm; when the wheel penetrated to the target depth of 10 μm at 2 μs, the crack depth increased to 84 μm. Thereafter, the propagation rate decreased significantly, and the crack depth stabilized at about 88 μm after 3 μs. The depth of the median crack was primarily determined during the penetration stage, with the subsequent scribing process having minimal influence, indicating that crack propagation occurs mainly within a very short period at the initial penetration phase.

### 3.2. Scribing Force

**Figure 4:**
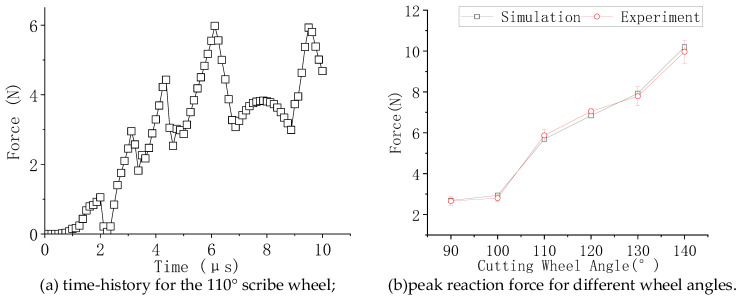
Reaction force at a scribing depth of 10 μm.

Figure 4a shows the reaction force during scribing. After the preset depth was reached at 2 μs, the reaction force continued to increase although the median crack depth had nearly stabilized, indicating that force evolution and crack-depth evolution are not fully synchronized. During the penetration stage, strong local stress concentration causes SPH particles along the median crack path to fail rapidly, so the median crack depth is mainly determined in this stage. After the damaged median-crack region is formed, part of the accumulated elastic energy is released through brittle fracture, and further growth of the median crack depth becomes limited. During the subsequent rolling stage, however, the wheel continues to compress and shear the near-surface and partially damaged glass material. This process causes stress redistribution, local compaction, intermittent brittle damage evolution, and possible lateral-crack tendency near the scribed surface. Therefore, the reaction force can continue to increase or fluctuate even when the median crack depth remains nearly stable. This phenomenon is consistent with previous studies showing that crack evolution in glass wheel scribing is a dynamic brittle-fracture process controlled by wheel geometry, contact load, local stress field, and material damage [15,17,18,19].

To evaluate experimental repeatability, three repeated tests were conducted for each wheel angle at the same preset scribing depth of 10 μm. The error bars in Figure 4b represent the standard deviation of the measured peak reaction force. The scatter may arise from slight wheel wear, local glass-surface variation, fixture compliance, and sensor fluctuation. Despite this scatter, the peak force increases consistently with wheel angle, in agreement with the simulation results.

**Figure 5:**
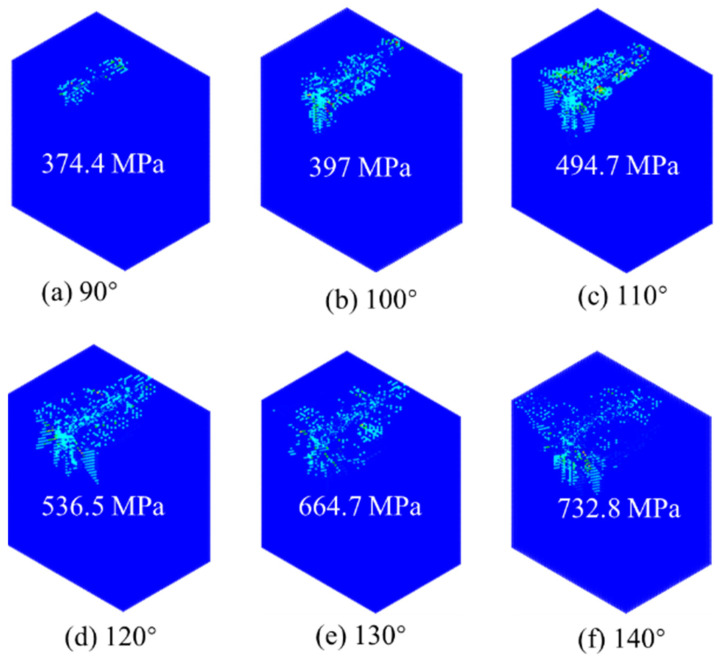
Effective stress distributions under different scribing wheel angles at t = 2 μs.

Based on the simulation model for the 110° scribing wheel, the SPH model was further extended to five additional scribing wheel angles: 90°, 100°, 120°, 130°, and 140°. These models were used to systematically investigate the influence of wheel angle on scribing behavior under a constant scribing depth of 10 μm. The maximum reaction force for each scribing wheel angle is presented in Figure 4b, and the corresponding effective stress distributions extracted at t = 2 μs are shown in Figure 5. This time corresponds to the end of the penetration stage, when the wheel reaches the preset depth of 10 μm; therefore, Figure 5 mainly reflects wheel-angle-dependent stress concentration and damage tendency during penetration.

Under the same scribing depth, the maximum effective stress for different scribing wheel angles is shown in the figure. As the wheel angle increases from 90° to 140°, the maximum effective stress rises from 374.4 to 732.8 MPa, corresponding to an overall increase of about 95.7%. This indicates that the effective stress generally increases with wheel angle, suggesting that the scribing wheel geometry has a significant influence on the internal stress response of the glass.

The increase, however, is not uniform across all angle intervals. In the range of 90–100°, the maximum effective stress increases only slightly from 374.4 to 397 MPa, indicating that the effect of wheel geometry on stress concentration is relatively limited at smaller angles. When the wheel angle increases to 110°, the maximum effective stress rises to 494.7 MPa, 24.61% higher than that at 100°, showing a much stronger growth trend. It further increases to 536.5 MPa at 120°, and then rises significantly to 664.7 MPa at 130°, with an increase of 23.89%, indicating that the stress response becomes more sensitive in this interval. At 140°, the maximum effective stress reaches 732.8 MPa, the highest among all cases.

In addition, as the wheel angle increases, the high-stress region becomes more concentrated and pronounced. This suggests that a larger wheel angle produces a stronger localized loading effect in the contact region, leading to more severe stress concentration in the near-scribing zone of the glass.

### 3.3. Median Crack Depth

**Figure 6:**
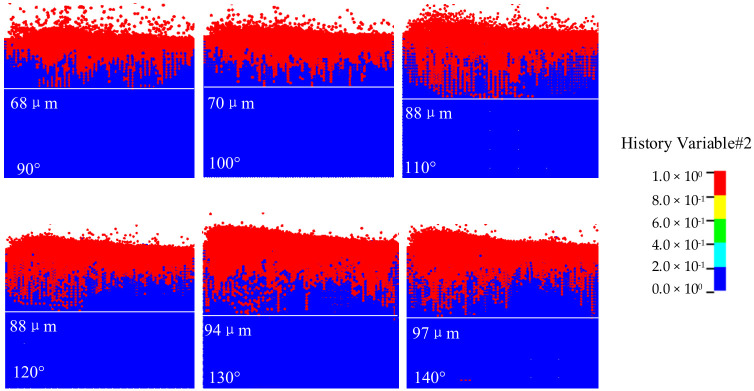
Simulated variation in median crack depth under different wheel angles.

According to the SPH simulation results, History Variable #2 was used to represent the damage variable D. When D reached 1, the corresponding particle was considered completely failed. The continuous region composed of failed particles with D = 1 was regarded as the simulated crack region, corresponding to the visible crack region in the experimental cross-section where material continuity was destroyed. The simulated median crack depth was defined as the vertical distance from the upper glass surface to the deepest failed particle connected to the median crack region. The median crack depths under different wheel angles are shown in Figure 6.

Figure 6 shows the simulated variation in median crack propagation depth under different scribing wheel angles. As the wheel angle increases from 90° to 140°, the median crack depth generally increases from 68 μm to 97 μm. The increase is relatively small in the range of 90–100°, whereas a pronounced rise is observed between 100° and 110°, indicating that this interval is particularly sensitive to the effect of wheel angle on crack propagation. Beyond 110°, the crack depth continues to increase, but the growth rate gradually decreases. These results suggest that increasing the scribing wheel angle promotes crack propagation into the thickness direction of the glass.

## 4. Experimental Validation

### 4.1. Experimental Setup

**Figure 7:**
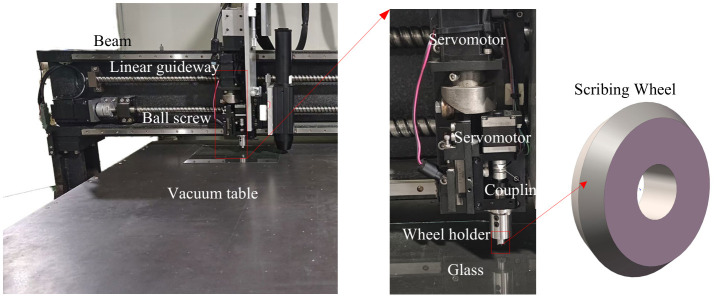
Self-developed glass scribing experimental platform.

The experimental validation was conducted on a self-developed glass scribing platform designed to reproduce practical scribing conditions as closely as possible, as shown in Figure 7. The system mainly consists of a vacuum adsorption stage with linear motion capability, a crossbeam, a precision lead screw, a linear guideway, servo motors, couplings, a support bracket, and a scribing wheel holder. During the experiments, the glass specimen was fixed on the vacuum adsorption stage to ensure motion stability and positioning accuracy, while the scribing wheel was mounted on the holder to perform the scribing operation. The coordinated motion of the stage and the transmission system enabled precise control of the scribing path and loading process. In this study, 0.2 mm thick Tianma LCD glass was selected as the experimental material, and a single-factor experimental method was employed to investigate the effects of scribing wheel angle and scribing pressure on crack formation behavior during glass scribing. The detailed experimental parameters are listed in Table 3.

To evaluate the repeatability of the experimental reaction force measurements, the scribing depth was set to 10 μm, and the reaction force required to reach this preset indentation depth was measured under different scribing wheel angles. For each wheel-angle condition, three repeated scribing tests were conducted under the same preset scribing depth. The average peak reaction force and standard deviation were then calculated. The error bars in Figure 4b represent the standard deviation of the three repeated measurements.

The scatter of the measured force may be related to slight wheel wear, local variation in glass surface condition, fixture compliance, and sensor fluctuation during the scribing process. Although minor scatter exists among repeated tests, the peak reaction force still shows a clear increasing trend with increasing wheel angle. This trend is consistent with the simulation results, indicating that the experimental measurements are repeatable and that the established model can reasonably capture the influence of wheel angle on the scribing force.

### 4.2. Experimental Force

The scribing wheel angle significantly affects the force required to reach the same scribing depth. As shown in Figure 4b, both simulation and experiment show that the peak reaction force increases with wheel angle from 90° to 140°. The experimental mean force increases from 2.66 N at 90° to 9.97 N at 140°, while the simulation results show the same trend. The maximum relative error is 5.17%, indicating that the model can reasonably predict the force variation.

The force changes only slightly from 90° to 100°, but increases sharply from 2.80 N to 5.87 N in the 100–110° interval, indicating stronger sensitivity of the force response in this range. Beyond 110°, the force continues to increase, but the growth is more gradual. Overall, larger wheel angles require higher force to achieve the same scribing depth, showing a nonlinear dependence of scribing force on wheel angle.

### 4.3. Median Crack Depth

**Figure 8:**
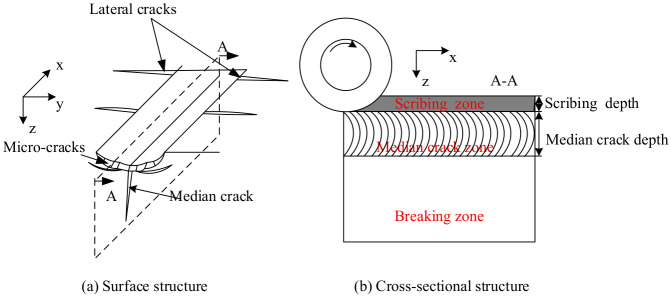
Schematic illustration of crack distribution on the surface and cross-section during glass wheel scribing: (**a**) surface; (**b**) cross-section.

The longitudinal cracks after wheel scribing are shown in Figure 8b. For the experimental measurement, the median crack depth was determined from cross-sectional microscopic images. Using the upper glass surface as the reference plane, the depth was measured along the thickness direction to the deepest visible tip of the median crack. This definition is consistent with the simulation, where the depth is measured from the upper surface to the deepest failed SPH particle with D = 1 connected to the median crack region.

**Figure 9:**
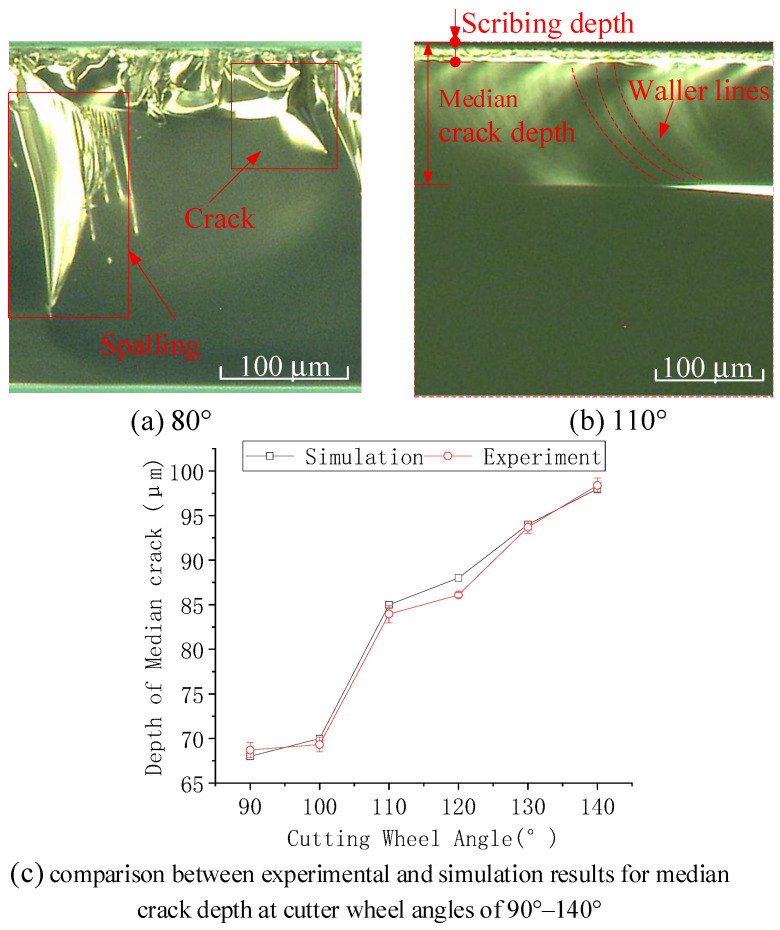
Cross-sectional morphology and median crack depth under different scribing wheel angles at a scribing depth of 10 μm: (**a**) 80°; (**b**) 110°; (**c**) comparison between experimental and simulation results for median crack depth at scribing wheel angles of 90–140°.

The cross-sectional morphologies and median crack depths under different wheel angles were analyzed to evaluate the influence of wheel angle on fracture behavior. When the scribing wheel angle was 80°, the cut cross-section exhibited disordered damage morphology, including irregular cracks, extensive spalling, and crack-opening regions, as shown in Figure 9a. Local ploughing-like scratches were also observed, indicating a scratching-dominated failure mode and unstable crack propagation at small wheel angles. By contrast, when the wheel angle was within 90–140°, the cross-sectional morphology was significantly improved, as shown in Figure 9b. Stable and continuous Wallner lines were observed, indicating more stable crack propagation and improved scribing uniformity. Therefore, the median crack depth in the range of 90–140° was further quantified under the same scribing depth of 10 μm, and the simulation results were compared with the experimental measurements, as shown in Figure 9c. The consistent variation trend between simulation and experiment indicates that the established model can reasonably capture the crack propagation behavior under different scribing wheel angles.

The scribing wheel angle has a significant effect on the median crack depth, and the simulation results agree well with the experimental results, showing a consistent variation trend. Within the range of 90–140°, both the simulation and experimental results indicate that the median crack depth increases with increasing scribing wheel angle. The maximum relative error between the simulation and experimental results is only 2.36%, demonstrating the reliability of the established model.

To evaluate the measurement uncertainty, three repeated scribing tests were conducted for each wheel-angle condition under the same preset scribing depth of 10 μm. The median crack depth was measured from cross-sectional microscopic images, and the average value and standard deviation were calculated. The error bars in Figure 9c represent the standard deviation of the three repeated measurements, with the standard deviation ranging from 0.27 μm to 1.00 μm.

Further analysis shows that the crack depth changes only slightly in the 90–100° range, with the experimental mean value increasing by 0.62 μm. The largest increase occurs in the 100–110° range, where the experimental mean value rises by 14.63 μm. Although some measurement scatter exists, this increase remains much more pronounced than that in the other adjacent angle intervals under the present experimental conditions. Therefore, the 100–110° interval can be regarded as a relatively sensitive range for median crack-depth growth. Beyond 110°, the crack depth continues to increase, but the growth rate generally decreases; the experimental mean values increase by 2.13 μm, 7.61 μm, and 4.68 μm in the 110–120°, 120–130°, and 130–140° ranges, respectively. These results indicate that increasing the scribing wheel angle promotes the extension of the median crack into the depth direction, with the most rapid increase occurring in the 100–110° interval.

### 4.4. Surface Morphology

**Figure 10:**
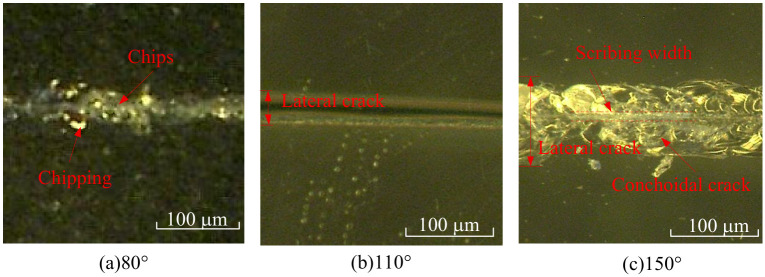
Surface morphologies of glass after scribing with scribing wheels of different angles: (**a**) 80°, (**b**) 110°, and (**c**) 150°.

Experimental results indicate that the scribing wheel angle has a significant influence on lateral cracking, edge chipping, and section quality during glass scribing. When the wheel angle is below 90°, obvious debris accumulation, pronounced edge chipping, and unstable sectional damage occur, as shown in Figure 10a. This suggests that a small wheel angle tends to induce severe local stress concentration and unstable fracture, resulting in poor crack controllability and surface quality. When the wheel angle is increased above 140°, obvious lateral cracks appear on the glass surface, as shown in Figure 10c. These lateral cracks may weaken the integrity of the scribed region and increase the risk of unstable fracture during subsequent breaking.

To further support the selection of the optimal scribing wheel angle, the scribing quality was evaluated by combining quantitative indicators with morphology-based observations. The reaction force and median crack depth were used to characterize the mechanical response and crack penetration capability, while chipping damage, lateral crack tendency, and section uniformity were evaluated from the surface and cross-sectional morphologies. Since Figure 10 provides representative microscopic observations rather than a complete statistical dataset for lateral crack length, lateral cracking was discussed based on its visible occurrence and severity instead of being reported as an absolute lateral crack range.

Based on this evaluation, the 110° scribing wheel shows a cleaner scribing groove, limited chipping damage, no obvious excessive lateral cracking in the observed region, and more uniform cross-sectional morphology with continuous Wallner lines. Combined with the quantitative results of reaction force and median crack depth, these morphology observations indicate that the 110° wheel provides the best balance among crack penetration, surface damage suppression, lateral crack control, and section uniformity under the present conditions. This recommendation is limited to 0.2 mm-thick LCD glass and a scribing depth of 10 μm. For other glass thicknesses, scribing depths, or material properties, the optimal angle may shift and should be further optimized.

## 5. Conclusions

(1) An SPH-based numerical model for LCD glass scribing with a scribing wheel was established, and its validity was verified experimentally through scribing force and median crack depth measurements. Under the same scribing depth of 10 μm, the maximum relative errors for reaction force and median crack depth prediction were 5.17% and 2.36%, respectively, indicating that the model can accurately characterize the mechanical response and crack propagation behavior during the glass scribing process.
(2) Crack propagation in glass exhibits distinct stage-dependent characteristics. The simulation results for the 110° scribing wheel show that median cracks are mainly initiated and rapidly propagate during the penetration stage. After the preset scribing depth is reached, the crack depth becomes nearly stable, and the subsequent rolling stage has little effect on it.
(3) The scribing wheel angle has a significant influence on scribing force, stress level, and median crack depth. Under the same scribing depth of 10 μm, as the scribing wheel angle increases from 90° to 140°, the scribing reaction force rises from 2.66 N to 9.97 N, corresponding to an increase of approximately 226.3%; the maximum effective stress increases from 374.4 MPa to 732.8 MPa, with an increase of about 95.7%; and the median crack depth increases from 68 μm to 97 μm. These results indicate that increasing the scribing wheel angle enhances local stress concentration in the scribing zone and promotes crack propagation along the thickness direction of the glass.
(4) The scribing wheel angle is a key parameter controlling scribing quality. Small angles tend to cause debris accumulation, edge chipping, and unstable section morphology, while large angles are more likely to induce lateral cracks. Under the present conditions of 0.2 mm-thick LCD glass and a scribing depth of 10 μm, the 110° wheel provides the best overall balance among median crack depth, surface damage suppression, lateral crack control, and section uniformity. This angle should be regarded as optimal only within the tested conditions, rather than as a universal value for all glass scribing processes.

## Figures and Tables

**Table 1:** Parameters of the Alloy Scribing Wheel.

Parameters	Values
Density *ρ* (kg·mm^−3^)	7.8 × 10^−6^
Young’s modulus E (GPa)	200
Poisson’s ratio μ	0.3

**Table 2:** JH-2 Model Parameters for Display Panel Substrate Material.

Parameters	Values
Density ρ (kg·mm^−3^)	2.53 × 10^−6^
Shear modulus, G (GPa)	26.9
A	0.71
B	0.178
C	0.0183
M	1
N	0.61
Hugoniot elastic limit HEL (GPa)	5.95
Hydrostatic pressure at the Hugoniot elastic limit PHEL (GPa)	2.92
Maximum normalized equivalent strength in undamaged state SFMAX	0.5
Maximum tensile hydrostatic pressure T (GPa)	0.0278
$D_{1}$	0.043
$D_{2}$	0.85
$K_{1}$	43.2
$K_{2}$	−67.2
$K_{3}$	153.2
Bulk constants	0.2

**Table 3:** Scribing parameters.

Parameters	Values
Scribing wheel angle (°)	80, 90, 100, 110, 120, 130, 140, 150
Scribing pressure (N)	2, 3, 4, 5, 6, 7, 8, 9, 10, 11

## Data Availability

The data and code are available from the corresponding author on reasonable request.
